# Comparison of Three Interview Methods on Response Pattern to Sensitive and Non-Sensitive Questions

**DOI:** 10.5812/ircmj.7673

**Published:** 2013-06-05

**Authors:** Ali Akbar Haghdoost, Mohammad Reza Baneshi, Sana Eybpoosh, Razieh Khajehkazemi

**Affiliations:** 1 Research Center for Modeling in Health, Kerman University of Medical Sciences, Kerman, IR Iran; 2 Regional Knowledge Hub for HIV/AIDS Surveillance, Kerman University of Medical Sciences, Kerman, IR Iran

**Keywords:** Sexual Behavior, Alcohols, Drug Users, Population

## Abstract

**Background:**

To get more precise responses when gathering information about sensitive topics such as drug use, it is important to use the most optimal method.

**Objectives:**

This study was carried out to address the impact of three interview methods (street-based, household, and telephone interviews) on response pattern to sensitive and non-sensitive questions in terms of participation, disclosure and discontinuing rates.

**Patients and Methods:**

We selected three culturally diverse major cities of Iran. Then, we randomly selected 300 subjects, 100 for each type of interview, from each major city (899 in total). For street-based interviews only pedestrians who were walking alone were recruited, for household interviews only one individual from each house participated (3-4 houses in each alley were selected), and for telephone interviews we selected phone numbers using a random number list. We asked five non-sensitive and five sensitive (related to drug use and sexual contact among their personal network) questions.

**Results:**

For telephone and household interviews, relative to street-based interviews, participants were less likely to disclose alcohol and drug-related behaviors (Adjusted OR = 0.76; 95% CI: 0.60- 0.97) and sexual behaviors among their network (Adjusted OR telephone/street-based = 0.64; 95% CI: 0.39- 1.07 and Adjusted OR household/ street-based = 0.56; 95% CI: 0.33- 0.95). We found that participants who were interviewed via the telephone were more likely (Adjusted OR = 1.24) and those who were interviewed at home were less likely (Adjusted OR = 0.86) to report non-sensitive information compared to participants who were interviewed on the street; however, these findings were not statistically significant. The largest participation rate and the least discontinuation rate were observed for household interviews.

**Conclusions:**

It seems that the methods of interview effect response to both sensitive and non-sensitive questions. We believe that for street-based interviews, respondents may disclose more sensitive information than telephone and household interviews.

## 1. Background

Collecting valid and high quality data about stigmatized and socially unacceptable behaviors is a major challenge (1). These can include drug abuse, sex-related issues, and even psychiatric problems. This is the case especially in countries in which these behaviors are treated as illegal or highly disrespected due to religious norms and values of that country. The basic problem with collecting information on these behaviors is that people are unwilling to disclose such information to the interviewer, sponsor organization of the survey, government, general public, friends or other household members and etc. (2, 3). If the mentioned issue is not addressed well in the study design, it could lead to bias in participant’s responses. There are some important aspects of data collection such as data collection mode (interview, self-administered), location (street, telephone, household, etc.) and question format (personalized versus depersonalized) that can influence the response of the respondent to a sensitive question (4). Different combinations of these factors could be applicable for the assessment of sensitive behaviors. Each combination varies in some characteristics such as the level of privacy and confidentiality that might be felt by the interviewee, the level of preparedness and concentration on the questions that are being asked, the proper comprehension of the questions, the burden of the sensitive question and etc. These variations can influence the accuracy and validity of the data (2); therefore, it is important to choose the best combination before running large-scale behavioral surveys. There have been a number of comparative studies throughout the world. For instance, Midanik (2003) reported that no difference in respondents’ answers to alcohol use was found between telephone and in-person interview methods (5); Pridemore et al. (2005) found that face-to-face interviews provide significantly more accurate information, when the primary interest is the recent use of alcohol and marijuana in welfare recipients. However, telephone interviews yielded higher or similar estimate of lifetime use of alcohol, marijuana, and drug, but underestimated the recent usage of these stuffs comparing to face-to-face interviews (6). Griesler et al. (2008) compared adolescents' self-reported smoking practice in two different settings (household vs. school); the results showed that adolescents tend not to report smoking practice in household interviews; this was prevalent especially among those whose social network comprised of non-smokers (7). Besides the controversial findings of the studies cited here, their results might not be applicable for other cultural settings because the response pattern to sensitive questions in each community is highly dependent on cultural context of that community. In Iran, there has been a number of investigations that have assessed sensitive behaviors in specific subgroups such as ecstasy use in university students (8), sexual experience among unmarried adolescent (9), and substance use among high school girls, and nursing students (10, 11) using self-administered questionnaires. Moreover, we found that some studies used face-to-face interviews to investigate the prevalence of high-risk behaviors (12). However, in our extended literature review, we have not found any study that compared different interview methods to find the best one for assessing high-risk behaviors, regarding Iran with a dominant Islamic culture. 

## 2. Objective

This study aimed to find which of the three interview methods (street-based, household, and telephone interview) yields the highest odds of information disclosure while asking sensitive questions. The results of this study might be used as a basis for future investigations in Iran and culturally similar communities that desire to use appropriate methods for exploring sensitive behaviors. 

## 3. Patients and Methods

This cross-sectional study was conducted during February and March 2011 in three main cities of Iran. We chose three culturally diverse cities of the country (including Tehran in the center of Iran with a mixture of communities and a population of more than 13.4 million, Urmia in north-west of Iran with a dominant Azari culture and a population of more than 2.8 million, and Kerman south-east of Iran with a dominant Fars culture and a population of more than 2.6 million (13) in order to achieve maximum possible variation in cultural mixture of participants. We prepared a comprehensive protocol and employed one supervisor for each city to manage and monitor the process. The supervisor, who must have lived in the selected city for at least the past 10 years, was selected among staff of Vice Chancellor for research of the medical universities in each city. Each supervisor selected two interviewers (one male and one female) among the members of student's research committee who should have had enough survey interviewing experience. The correspondent supervisor selected three crowded areas of the city that had the most economical and social variations. In each area, three types of interviews (street-based, household, and telephone interview) were performed within a short period of time. All interviewers received the comprehensive protocol that they were supposed to follow and the interviewer guideline which included the legitimacy and importance of the survey.

### 3.1. Sample Selection in Each Main City

From each main city, a random sample of 300 dwellers were selected (total sample size was 900). The estimated sample size in each area was 100; therefore, 30-35 participants were allocated to each mode of interview. Interviewers were supposed to recruit a relatively equal proportion of both genders in each area and for each type of interview. All interviews were performed during 9-11 A.M. and 4-7 P.M. The interviewers and participants were gender-matched. To be eligible for the study, participants should have been aged at least 18 years old and have lived in the selected city for at least the past five years. 

### 3.2. Street-based Interviews

In each area, some crowded streets were selected. In order to provide privacy and prevent bias in participants’ response, only pedestrians who were walking alone were approached. 

### 3.3. Household Interviews

In each area, at least ten alleys were randomly selected. In each alley only 3-4 houses were selected as well as only one house in each apartment and one person from each house (who met the eligibility criteria). To avoid duplication in selection of an alley or an apartment/house, both interviewers were asked to be together during selection of households. Once again, in order to provide privacy and prevent bias, each participant was interviewed alone by one interviewer.

### 3.4. Telephone Interviews

We obtained the pre-number dialing code of each area from the telecommunication company; the remaining digits of each phone number were generated by means of the table of random numbers. All interviewers were instructed to use the table of random numbers. Non-working (unassigned) and non-residential telephone numbers (e.g., office numbers), residential numbers which were used only for non-voice purposes (e.g., facsimile machine), and telephone numbers for which the eligible person was not at home, were excluded. Moreover, each phone number was only dialed once. Interviews were conducted with the first eligible person who answered the telephone; if that person was not eligible, the interviewer asked for another person in that household (who met the eligibility criteria) for interview.

### 3.5. Measurements

We used a short data collection form, mainly extracted from the questionnaire of a big national study titled as “Size estimation of alcohol and drug users in I. R. Iran”(14), that was made up of four main parts. The first two parts covered information about demographic characteristics and active social network size of the respondent. The two remaining parts included questions that covered five non-sensitive and five sensitive behaviors among their active social networks [See Appendix 1]. The interviewer asked the questions and recorded respondent’s answers. Content validity of this form was confirmed by taking into consideration the viewpoints of two professors that had experience in Network Scale Up (NSU) surveys in Iran.

### 3.6. Statistical Analysis

For each city, differences in demographic characteristics between three interview methods were assessed using one-way ANOVA (for continues variables) and Pearson Chi-square (for categorical variables). Logistic regression model, using backward stepwise method, was used to assess the impact of method of interview on disclosing sensitive and non-sensitive information. In this regard, the first full model included the interaction terms between method of interview and city of residence, along with demographic characteristics measured at enrollment. Likelihood Ratio Test (LRT) was used to compare different models. We calculated Adjusted Odds Ratios (AOR) with 95% confidence intervals (CI), controlling for sex, age, marital status, education and city of residence. Due to the nature of questions and level of their sensitivity in the context of Iran, we classified the questions into three categories: (I) non-sensitive questions, (II) alcohol and drug-related questions, and (III) sexual questions. Random effects logistic regression model was fitted on categories I and II; for category III, logistic regression model was used. All statistical analyses were performed using the SPSS software (Ver. 10.0). A P value of < 0.05 was considered as statistically significant.

### 3.7. Ethical Consideration

At enrollment, after all participants were assured about the confidentiality of their responses and anonymity of their participation, verbal informed consent was obtained. Moreover, participants were informed that they could leave the interview whenever they wanted (in this occasion, the interviewer was supposed to insert the reason of discontinuation in the data collection form). This study was approved by the Ethics Committee of Kerman University of Medical Sciences.

## 4. Results

### 4.1. Participation Rate 

The participation rate varied by interview method (P = 0.028). This rate was 68.8% (300 out of 436 eligible dwellers) and 77.1% (300 out of 389 eligible dwellers) for street-based and household interviews, respectively. After calling 2272 phone numbers, 299 telephone interviews were completed; as there was 112 eligible dwellers that did not consent to participate in the study, the participation rate for telephone interviews was 72.7% (299 out of 411 eligible dwellers). 

### 4.2. Demographic Characteristics

A total of 899 (468 female and 431 male) participants were recruited. Table 1 presents the distribution of demographic characteristics of participants for different combinations of methods of interview by the city of residence. Mean ± SD age of all studied participants was 35.7 ± 13.2 years; with Inter Quartile Range (IQR) between 25 and 44 years. Overall, most respondents were married/ ever married (64.5%) and had a Diploma/ under Diploma level of education (60.7%). This pattern was observed consistently in Tehran and Urmia. However, in Kerman the pattern was somewhat different. In a way that street-based interviews in Kerman was conducted on those who were mostly single (54%) and had an upper Diploma level of education (54.5%). Moreover, in Kerman and Urmia, street-based interviews were carried out on younger dwellers (the means of age were 26.3 and 32.6 years, respectively). There was a significant difference in age, marital status and educational level of participants between the three interview methods in Kerman (all P < 0.0001); however, in Urmia only participants’ mean age showed a significant difference between the three interview methods (P < 0.0001).

**Table 1.** Demographic characteristics of study participants of three interview methods by city

Demographic Characteristics	Mode of Interview Administration								
	Kerman			Tehran			Urmia		
	S ^c^ (n = 101)	T ^c^ (n = 100)	H ^c^ (n = 99)	H ^c^ (n = 101)	T ^c^ (n = 100)	S ^c^ (n = 99)	H ^c^ (n = 100)	T ^c^ (n = 99)	S ^c^ (n = 100)
**Age, y, Mean ± SD**	39.4 ± 15.2	39.0 ± 13.	26.3 ± 6.5	37.8 ± 12.6	34.8 ± 11.2	34.8 ± 11.2	33.1 ± 10.9	43.3 ± 16.2	32.6 ± 12.2
**P value^a^**	< 0.0001			0.107			< 0.0001		
**Marital status, No. (%)**									
Single	32 (32.0)	20 (21.3)	36 36.0)	43 (42.6)	41 (41.0)	39 39.4)	28 (28.6)	23 (23.5)	54 (54.0)
Married/ever married	68 (68.0)	74 (78.7)	64 (64.0)	58 (57.4)	59 (59.0)	60 (60.6)	70 (71.4)	75 (76. 5)	46 (46.0)
**P value^b^**	< 0.0001			0.901			0.07		
**Education, No. (%)**									
Diploma/ under Diploma	63 (63.0)	51 (51.5)	62 (62.0)	69 (68.3)	71 (71.0)	58 (58.6)	69 (69.7)	57 (57.0)	46 (45.5)
Upper Diploma	37 (37.0)	48 (48.5)	38 (38.0)	32 (31.7)	29 (29.0)	41 (41.4)	30 (30.3)	43 (43.0)	55 (54.5)
**P value^b^**	0.003			0.151			0.190		

^a^Computed by One-way ANOVA

^b^Computed by Chi-squared test

^c^Abbreviations: H, Household interview; S, Street-based interview; T, Telephone interview

### 4.3. Disclosure of Sensitive Information 

**Figure 1.**
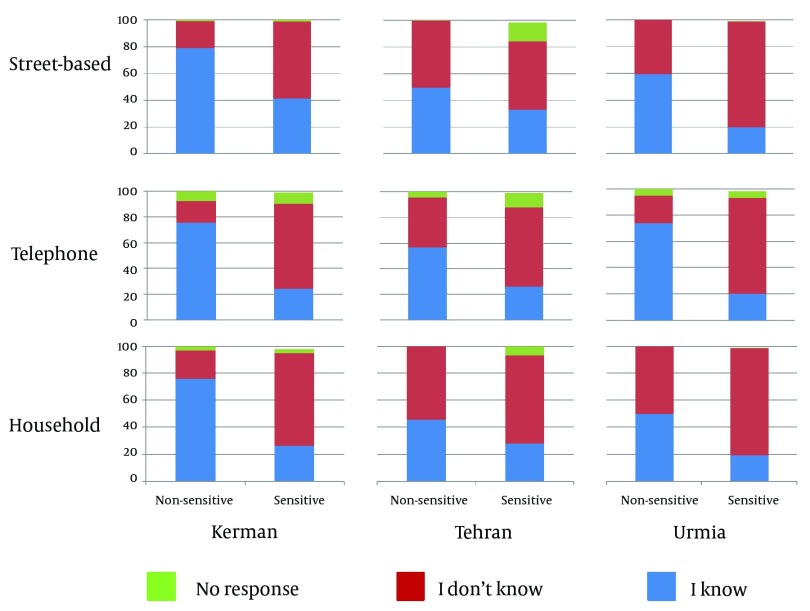
Disclosure Rate (%) of Sensitive and Non-sensitive Information by City and Type of Interview

Figure 1 presents disclosure rate of sensitive and non-sensitive information of different data collection methods classified by city. Crude analysis showed that street-based interviews in Tehran and Kerman lead to the highest disclosure rate of sensitive information; however, there was no difference between the three types of interviews in Urmia. Moreover, disclosure rate of alcohol and drug-related information (group I) was similar to sexual information (group II). More detailed analysis showed that participants who were interviewed via the telephone or at their houses had a significantly lower rate of disclosing sensitive information than those who were interviewed on the street when asked about alcohol and drug-related questions (AOR telephone/street-based and AOR household/ street-based = 0.76; 95% CI: 0.60- 0.97) or sexual behaviors in their network (AOR telephone/street-based = 0.64; 95% CI: 0.39- 1.07 and AOR household/ street-based = 0.56; 95% CI: 0.33- 0.95). Participants with upper diploma, were more likely to disclose stigmatized behaviors in their social network rather than those with diploma or lower levels of education (AOR in group I = 1.57; 95% CI: 1.28- 1.92 and AOR in group II = 1.90; 95% CI: 1.22- 2.97). In other words, the former group was more likely to know someone who has engaged in these behaviors during the past two years (Table 3). We found that, the nature of the questions was the frequent reason for stopping the interview in each interview mode. However, in telephone interviews, about 35% of respondents stopped the interview because of reasons other than the sensitive nature of the question (Table 2). 

### 4.4. Disclosure of Non-sensitive Information 

Considering Kerman and Tehran, crude analysis showed that disclosure rate of non-sensitive information was the same within the three interview methods; however, this similarity was not seen in Urmia which showed a higher rate of non-sensitive information disclosure for telephone interviews compared to the other two methods (Figure 1). More detailed analysis showed that participants who were interviewed via the telephone were more likely and those who were interviewed at home were less likely to disclose non-sensitive information compared to participants who were interviewed on the street, however, these findings were not statistically significant (AOR telephone/street-based = 1.24; P-value = 0.940; AOR household/ street-based = 0.86; P-value = 0.418). Those who had higher levels of education and married participants were more likely to disclose non-sensitive information (Table 3). 

**Table 2.** Distribution of the Reason for Discontinuation of the Interview, by Interview Mode

Reason of Discontinuing the Interview^a^	Street-based (n = 33), No. (%)	Telephone (n = 30), No. (%)	Household (n= 22), No. (%)
**Having no time**	1 (3.0)	3 (10.0)	1 (4.5)
**Nature of questions**	29 (87.9)	16 (53.3)	19 (86.4)
**Others**	3 (9.1)	11 (36.7)	2 (9.1)

^a^Note: There was a possibility of choosing more than one item

**Table 3.** Odds of Disclosing Sensitive and Non-Sensitive Information by Mode of Interview, City Of Residence, and Demographic Characteristics

Information	Type of Sensitive Question				Non-sensitive Questions	
	Alcohol and Drug-Related Questions		Sexual Questions			
	AOR (95% CI)	P value	AOR (95% CI)	P value	AOR (95% CI)	P value
**Mode of interview**						
Street-based	Ref	---------	Ref	---------	Ref	---------
Telephone	0.76 (0.59, 0.97)	0.027	0.64 (0.39, 1.07)	0.091	1.02 (0.70, 1.50)	0.904
Household	0.76 (0.60,0.97)	0.026	0.56 (0.33, 0.95)	0.033	0.86 (0.59, 1.24)	0.418
**City of residence**						
Kerman	---------	Ref	---------	Ref	---------	Ref
Tehran	1.02 (0.81, 1.29)	0.860	1.26 (0.77, 2.07)	0.350	0.22 (0.15, 0.31)	< 0.0001
Urmia	0.53 (0.41, 0.67)	< 0.0001	0.42 (0.24, 0.76)	0.004	0.35 (0.25, 0.50)	< 0.0001
**Interaction terms**						
Telephone, Tehran	---------	---------	---------	---------	1.59 (0.97, 2.61)	0.067
Household, Tehran	---------	---------	---------	---------	2.02 (1.21, 3.37)	0.007
Telephone, Urmia	---------	---------	---------	---------	0.99 (0.61, 1.60)	0.970
Household, Urmia	---------	---------	---------	---------	0.73 (0.45, 1.19)	0.207
**Sex (male/ female)**	1.51 (1.24, 1.84)	<0.0001	---------	---------	0.89 (0.75, 1.04)	0.134
**Education (upper Diploma/ under Diploma)**	1.57 (1.28, 1.92)	< 0.0001	1.90 (1.22 , 2.97)	0.005	1.61 (1.36, 1.91)	< 0.0001
**Marital status (Married & Ever married/ single)**	---------	---------	0.60 (0.38, 0.93)	0.024	1.20 (0.98, 1.48)	0.078
**Age,y**	0.99 (0.98 , 1.00)	0.079	---------	---------	1.01 (1.00, 1.02)	0.036

## 5. Discussion

Our results indicated that the type of interview influences the disclosure pattern of both sensitive and non-sensitive questions. Regarding sensitive questions, participants who were interviewed on the street had a greater likelihood of disclosing information about sexual, alcohol and drug-related questions compared to those who were interviewed via the telephone or at their home. Considering non-sensitive questions, disclosure was higher for street-based interviews rather than household or telephone interviews. Our result is consistent with previous studies that have concluded that the mode affects disclosure of information (especially sensitive questions) (15-17). We believe that participants who have been interviewed on the street might be more relaxed about disclosing information because they might be conceived that their selection has been done completely by chance and their participation is really anonymous, therefore, their responses will be kept confidential. In contrast, those who are being interviewed at home or via the telephone might be doubtful about the random nature of their selection and anonymity of their responses because the interviewer knows their home address or phone number. This fact can make them feel uncomfortable or frightened, especially for disclosing sensitive information. Paulhus (1984) states that the more respondents feel assured of the anonymity, the more honest their responses tends to be (18). As the social desirability also biases the answers (17), depersonalized questions were chosen in this study. In this method people are asked about stigmatized or sensitive behaviors in their network rather than themselves; the method which is the key concept in network scale up technique in estimation of the size of hidden sub-populations (19). Furthermore, we found that street-based interviews have led to the lowest participation rate. Therefore, street-based interviews may be better to obtain more accurate responses while extracting sensitive, illegal, or stigmatized information is the main priority rather than the proportion of interviewees who consent to take part in the study. In this study, the pattern of sensitive information disclosure in different ethnic groups and cities was the same. However, the rate of non-sensitive information disclosure in Urmia was different from Kerman and Tehran. In Urmia, the interviewers were chosen among Azeri people and based on interviewers’ claim, most interviews were conducted in the Azeri language (the local language of Urmia people); therefore, interviewers ought to translate the questions to Azeri for the interviewee; this might change the meaning of the question entirely or in part and might be the source of change in our results. Therefore, researchers should consider language differences adherent to the community they are studying. Discontinuation of the interview due to the sensitive nature of the questions happened more frequently in Urmia. People’s sensitivity to stigmatized issues and their reaction to feelings of threat might differ by sub-cultures of each community as well as respondents’ personal characteristics. Therefore, characteristics of each community’s sub-cultures should be considered while designing such studies. On the other hand, Dotinga, et al. (2005) concluded that Turks and Moroccans who were interviewed by a Dutch interviewer (non-ethnically-matched interviews) were significantly three times more likely to report their alcohol use rather than Turks and Moroccans who were interviewed by a Turk or Moroccan interviewer (ethnically-matched interviews) (20). Krysan and Couper (2006) also reported that race of the interviewer (black vs. white) affects the responses of interviewees (21). As mentioned above, in Urmia, the interviews were conducted in the Azeri language (local language of Urmia people); therefore, high rate of discontinuation of interview may be due to the fact that in Urmia the interviewees have felt a closer connection with interviewers because they spoke in the local language of city’s residents (Azeri language) but not in the formal language of the country and thus the participants might be afraid of being known by the interviewer. In Kerman and Urmia the interviewers included younger dwellers in street-based interviews. It is more probable that younger participants recall their acquaintances better than aged ones; this could bias the results of the second part of the questionnaire (see Measurements section). Moreover, younger respondents may differ with older ones in their level of caution, patience, and interest; this could bias the results of sensitive and non-sensitive information disclosure (part three and four of the questionnaire, see Measurements section). In addition, our interviewers were all young adults as well. Lin et al. (1992) studies the effect of age and sex similarity between interviewers and interviewees and concluded that age similarities do not affect interview outcomes however race similarities do (22). While there could be numerous combinations of data collection methods based on mode and location of data collection, question format, etc., we only compared three combinations (including street-based, household, and telephone-based interview methods); however, these combinations were considered in this study because they were commonly used, were relatively cost-effective, (23, 24) and could be applicable for large-scale investigations (25); further studies seem to be essential to evaluate the effectiveness of other interview methods as well as self-administered, internet-based, or any other techniques in obtaining valid and trustworthy results. In this regard, comparing the responses received via these methods with those obtained within in-depth interview methods might be helpful in assessing the validity and trustworthiness of the responses. We only selected three cities, and asked about the limited number of sensitive and non-sensitive questions. Further investigations will provide better insights about the applicability of these methods in other cities and sub-cultures of Iran.
